# Patient-Reported and Oncological Outcomes of Salvage Therapies for PSMA-Positive Nodal Recurrent Prostate Cancer: Real-Life Experiences and Implications for Future Trial Design

**DOI:** 10.3389/fonc.2021.708595

**Published:** 2021-06-21

**Authors:** Alexander Kretschmer, Johanna Milow, Chukwuka Eze, Alexander Buchner, Minglun Li, Thilo Westhofen, Frederik Fuchs, Paul Rogowski, Christian Trapp, Ute Ganswindt, Mathias Johannes Zacherl, Leonie Beyer, Vera Wenter, Peter Bartenstein, Boris Schlenker, Christian G. Stief, Claus Belka, Nina-Sophie Schmidt-Hegemann

**Affiliations:** ^1^ Department of Urology, LMU Klinikum, Ludwig-Maximilians University Munich, Munich, Germany; ^2^ Department of Radiation Oncology, LMU Klinikum, Ludwig-Maximilians University Munich, Munich, Germany; ^3^ Department of Therapeutic Radiology and Oncology, Innsbruck Medical University, Innsbruck, Austria; ^4^ Department of Nuclear Medicine, LMU Klinikum, Ludwig-Maximilians University Munich, Munich, Germany

**Keywords:** prostate cancer, PROMS, PSMA - prostate specific membrane antigen, salvage lymph node dissection, salvage radiotherapy

## Abstract

**Introduction:**

The role of salvage lymph node dissection (SLND) and radiotherapy (SLNRT) in the management of nodal-only recurrent prostate cancer (PC) remains controversial. In addition, impact on health-related quality of life (HRQOL) has not been adequately evaluated yet.

**Materials and Methods:**

Analysis was limited to patients that were diagnosed with nodal-only recurrent PC *via* PSMA-PET/CT. SLND was performed *via* open approach. For SLNRT, dose regimens were normo- or slightly hypo-fractionated with a simultaneous boost to the PET-positive recurrences. EORTC QLQ-C30 and PR-25 questionnaires were used to assess HRQOL. Continence status was assessed using daily pad usage and the validated ICIQ-SF questionnaire. For multivariable analysis, Cox regression models were used (p<0.05).

**Results:**

138 patients (SLND: 71; SLNRT: 67) were included in the retrospective analysis. Median follow-up was 47 months (mo) for SLNRT patients (IQR 40–61), and 33mo for SLND patients (IQR 20–49; p<0.001). In total, 61 patients (91.0%) in the SLNRT cohort and 43 patients (65.2%; p<0.001) in the SLND cohort underwent ADT anytime during the follow-up period. In multivariate Cox regression analysis, SLNRT could be confirmed as an independent predictor for increased PSA progression-free survival (PFS; HR 0.08, 95%CI 0.040 – 0.142, p<0.001). Estimated median metastasis-free survival (MFS) was 70mo for the total cohort without statistically significant differences between both subgroups (p=0.216). There were no significant differences regarding general HRQOL, daily pad usage, and ICIQ-SF scores between the respective cohorts.

**Conclusions:**

In a large contemporary series of patients with nodal-only recurrent PC based on PSMA-PET/CT staging, we observed significantly increased PSA PFS in patients undergoing SLNRT while no significant differences could be observed in MFS, and functional outcomes including HRQOL.

## Introduction

Clinical recurrence based on PSA progression and/or imaging findings can occur in up to 20% of patients that underwent radical prostatectomy (RP) due to prostate cancer (PC) (1). While it has been shown that nodal recurrences have a better prognosis compared to patients with bone or visceral metastases (2), the optimal treatment regimen of these patients is still a matter of debate. With novel imaging modalities such as PSMA-PET being implemented in routine diagnostic protocols in these clinical scenarios, nodal-only recurrences are more frequently diagnosed (3). Salvage lymph node dissection (SLND) has shown some promising results (4) and recent evidence from multi-institutional retrospective studies suggest to implement SLND in a multimodal therapeutic concept (5). Salvage lymph node radiotherapy (SLNRT) is an alternative treatment modality for nodal-only recurrent PC. Hereby, stereotactic body radiotherapy and elective nodal radiotherapy have been described as possible options in a recent systematic review (6). While retrospective, non-comparative data suggests a wide range of progression free survival rates (4, 6), comparative data are still rare. Evidence is further hampered by the fact that different radiation strategies, surgical templates and androgen deprivation therapy (ADT) regimens have been used and that staging has been based on various imaging diagnostics. In preliminary findings with limited sample size and follow-up by our working group, we found significantly improved PSA progression-free survival (PFS) for patients that underwent SLNRT instead of SLND for PSMA-PET/CT-based nodal recurrent PC (7).

In addition, since long-term oncologic benefits remain unclear, assessment of the impact of these treatment modalities on health-related quality of life (HRQOL) is crucial. In the current study, we provide oncological and patient-reported outcomes with a special focus on HRQOL of a contemporary cohort of patients that underwent metastasis-directed salvage therapies for PSMA-PET/CT-based nodal recurrent PC.

## Materials and Methods

### Patient Population, Definitions and Study Design

For the current analysis, consecutive patients who underwent SLND or SLNRT due to PSMA-PET/CT-based nodal-only recurrence of prostate cancer at a single tertiary care academic center between 2014 and 2019 were included. All patients had a history of radical prostatectomy and included patients with or without measurable PSA levels post-RP (**Table 1**). Patients with previous radiotherapy of pelvic nodes or previous androgen deprivation therapy (ADT) were excluded from further analysis. Decision of SLNRT or SLND was based on patient’s preference.

**Table 1:** Patient characteristics of 138 patients that underwent further analysis (ADT, androgen deprivation therapy; IQR, interquartile range; PSA, prostate specific antigen; RP, radical prostatectomy; SLND, salvage lymph node dissection; SLNRT, salvage lymph node radiotherapy).

	SLND (N=71; 51.4%)	SLNRT (N=67; 48.6%)	P
**Median PSA at RP** [ng/ml; (IQR)]	10.9 (6.9 - 19.3)	17.3 (8.2 - 32.4)	0.045
**Pathologic tumor stage at RP** [n (%)]			
pT2	22 (31.0)	12 (17.9)	<0.001
pT3a	38 (53.5)	18 (26.9)	
pT3b	11 (15.5)	35 (52.2)	
pT4	0 (0.0)	2 (3.0)	
**Pathologic ISUP group at RP** [n (%)]			
≤2	18 (25.4)	12 (17.9)	0.310
3-5	53 (74.6)	55 (82.1)	
**Nodal status at RP** [n (%)]			
pN0	41 (57.7)	36 (53.7)	0.009
pN1	20 (28.6)	30 (44.8)	
pNx	10 (14.3)	1 (1.5)	
**Positive surgical margins at RP** [n (%)]			
No	48 (67.6)	31 (46.3)	0.015
Yes	23 (32.4)	36 (53.7)	
**Undetectable PSA after RP** [n (%)]			
No	34 (47.9)	50 (74.6)	0.028
Yes	37 (52.1)	17 (25.4)	
**ADT anytime during follow-up** [n (%)]			
No	28 (34.8)	6 (9.0)	<0.001
Yes	43 (65.2)	61 (91.0)	
**Median PSA at salvage treatment** [ng/ml; (IQR)]	2.6 (1.3 - 5.6)	1.3 (0.6 - 3.7)	0.008
**Median time RP to salvage treatment** [mo; (IQR)]	25 (4 - 47)	6 (4 - 26)	0.116

After approval of a local ethics committee (LMU #18-020), questionnaires were sent to patients who fulfilled the inclusion criteria in October 2020 as part of a cross-sectional analysis. As per institutional standards (8), urinary continence was assessed using the International Consultation on Incontinence Questionnaire in its short form (ICIQ-SF). The ICIQ-SF is a three-item validated questionnaire. The total score ranges from 0 to 21, with higher scores indicating greater severity of urinary incontinence (9). HRQOL was assessed using the European Organization for Research and Treatment of Cancer quality of life questionnaires (EORTC QLQ) C30 and PR25. General health-related quality of life (HRQOL) was assessed using the global health status domain of the QLQ-C30 questionnaire (questions 29 and 30) following current EORTC instructions (10). Following Snyder et al., good general HRQOL is defined as global health status of ≥70 (11). In general, higher scores for global health status represent better general HRQOL. Erectile dysfunction was assessed *via* the validated International Index of Erectile Function (IIEF5) questionnaire. PSA persistence was defined as a minimum PSA level of 0.2 ng/ml for SLND and SLNRT patients. Follow-up time was defined as the interval in months between SLND/SLNRT and the last recorded PSA. Biochemical recurrence was defined as first measurable PSA >0.2ng/ml after salvage treatment irrespective of ADT.

Follow-up examination was first performed six weeks to three months after SLND/SLNRT and then every six to 12 months.

### Radiotherapy

All patients received elective nodal radiotherapy (ENRT) by intensity-modulated radiotherapy (IMRT) or volumetric arc therapy (VMAT) and image-guided radiotherapy techniques (IGRT, 2-5 times/week). Radiotherapy dose regimens were normo- or slightly hypo-fractionated and a boost to the PET-positive local recurrences within prostatic fossa and lymph nodes was applied simultaneously. Planning CT was done in supine position. Patients were advised to have a full bladder and empty rectum. Target delineation was performed according to the Radiation Therapy Oncology Group (RTOG) atlas for salvage PC and for lymphatic pathway delineation (12).

### Salvage Lymph Node Dissection

The standard SLND procedures at our department have been described before (3, 13). Briefly, an open approach through an abdominal midline incision was used and standard extended SLND was performed based on specific regions according to the most recent PSMA-PET/CT findings. No radio-guided surgery has been performed. Dissected lymph nodes were classified based on the respective anatomic region. Routinely, dissected lymph nodes were immediately sent for histopathologic analysis and evaluated according to standard protocols (serial sectioning, 200 μm slices). Perioperative complications were defined as complications that occurred intraoperatively or up to 90d postoperatively and were graded using the Clavien Dindo classification (14).

### Statistical Analysis

Primary endpoint was PSA progression-free survival (PFS) and was defined based on a PSA cut-off level of ≥0.2 ng/ml. Demographic data were analyzed using descriptive statistics and χ2 test. For survival analysis, Kaplan Meier analyses and log-rank testing were performed. For multivariate analysis, Cox regression models accounting for significant differences in patient as well as treatment characteristics were used. A p value of <0.05 was considered statistically significant. For statistical analysis, SPSS Statistics 27 (IBM, Armonk, NY, USA) was used.

## Results

### Patients Characteristics and Safety

In total, 138 patients (SLND: 71; SLNRT: 67) were included in the current analysis. Basic patient characteristics are summarized in **Table 1**. Briefly, a general pattern towards more aggressive disease in terms of median PSA at the time of RP (p=0.045), pT stage (p<0.001), nodal status (p=0.009), and positive surgical margin rate (p=0.015) were observed for the SLNRT cohort. Median PSA at salvage treatment was 1.3 ng/ml (IQR 0.58 – 3.65) for SLNRT patients and 2.56 (IQR 1.32 – 5.63; p=0.008) for SLND patients.

Median follow-up was 47 months for the SLNRT cohort (IQR 40 – 61), and 33 months for the SLND cohort (IQR 20 – 49; p<0.001).

Regarding the SLND cohort, 41 patients (57.7%) had undergone radiation of the prostatic bed.

At PSMA-staging following post-RP biochemical recurrence, a median number of 2 lymph nodes (IQR 1 – 3) were suspect in PSMA scans. Hereby, suspect lymph nodes were located in the pelvic region in 67.1%, retroperitoneal in 14.3% and in both regions combined in 18.6%. A median number of 9 (IQR 1 – 44) lymph nodes were removed with a median number of histologically proven prostate cancer positive lymph nodes of one (IQR 0 – 16). 27 of the SLND patients (38.0%) underwent bilateral SLND, whereas pick-up-SLND was performed in 13 patients (18.3%). In a total number of 20 patients (28.2%), less tumor-positive lymph nodes than preoperative PSMA-positive lesions were detected. Furthermore, no tumor-positive lymph node was detected in 10 patients (14.1%).

As depicted in **Supplementary Table 1**, perioperative complications were observed in 17 patients (23.9%). Most frequent complication was symptomatic lymphocele that required percutaneous drainage. Clavien grade 3 or 4 complications occurred in 7 patients (9.9%).

For SLNRT patients, median number of PSMA-positive lymph nodes was 2 (IQR 1 – 4). Suspect lymph nodes were pelvic only in 80.6%, retroperitoneal only in1.5% and in both regions combined in 17.9%. Patients received radiotherapy treatment as depicted above. Sixty patients had the prostatic fossa treated with a median number of 66 Gy (range, 60 - 67.20 Gy) in single doses of 2 Gy (range, 1.8 - 2.12 Gy). All patients received radiotherapy of the elective lymphatic pathways with 50.4 Gy (range, 45.0 - 52.3 Gy) in single doses of 1.8 Gy (range, 1.6 - 1.8Gy). PSMA PET-positive local recurrences within in the prostatic fossa, diagnosed in 15 patients, were irradiated with a median number of 70.0 Gy (range, 68 - 70 Gy). PSMA PET-positive lymph nodes were treated with a median number of 61.6 Gy (range, 50.4 - 66 Gy).

Acute grade 2 gastrointestinal and urogenital toxicity were each observed in 19 patients (28.4%) consisting primarily of diarrhea and increased urinary frequency. Acute grade 3 urogenital toxicity occurred in one patient (1.5%) with evidence of urethral stenosis. Late Grade 2 toxicity was overall seen in 24 patients (35.8%) with mainly signs of erectile dysfunction and increased urinary frequency. Late grade 3 toxicity was present in 25 patients (37.3%) with erectile dysfunction (36%) being the most frequently observed (**Supplementary Table 2**).

### Peri- and Postinterventional Androgen Deprivation Therapy

Periinterventional ADT was administered in 60 patients (89.6%) in the SLNRT subgroup, compared to 4 patients (5.6%; p<0.001) in the SLND subgroup.

Furthermore, a total number of 61 patients (91.0%) in the SLNRT cohort and 43 patients (65.2%; p<0.001) in the SLND cohort underwent ADT anytime during the entire follow-up period. Median ADT duration for SLNRT patients was 9 months (IQR 3 – 21).

Consequently, 13 patients in the SLNRT subcohort (19.4%) compared to 43 patients (65.2%; p<0.001) in the SLND cohort were under active ADT at the individual maximum follow-up time point.

### Functional Outcomes and Health-Related Quality of Life

HRQOL outcomes based on the validated EORTC QLQ-C30 questionnaires and the EORTC PR-25 add-on are summarized in **Table 2**. Briefly, significantly increased appetite loss scores [9.8±20.7 (SLNRT: median 0, IQR 0 - 0) *vs.* 0.9±5.5 (SLND; median 0, IQR 0 - 0); p=0.019] and diarrhea scores [22.5±25.2 (SLNRT; median 33.3, IQR 0 – 33.3) *vs.* 13.0±25.2 (SLND; median 0, IQR 0 – 8.3); p=0.038] were found for patients in the SLNRT subcohort. There were no significant differences regarding the QLQ-C30 functioning scores as well as general HRQOL based on the QLQ-C30 global health status. Regarding previously described cut-off values (11), 63.6% of the patients in the SLNRT cohort compared to 58.3% in the SLND cohort stated good general HRQOL (p=0.805). In addition, no statistically significant differences regarding the PR-25 items existed, except for the incontinence aid score which was significantly increased in the SLNRT cohort [43.3±26.0 (SLNRT; median 33.3, IQR 0 – 45.8) *vs.* 24.1±29.0 (SLND; median 16.7 (0 – 33.3); p=0.035].

**Table 2:** Health-related quality of life outcomes based on the validated QLQ-C30 and QLQ-PR25 questionnaire.

		SLNRT	SLND	p
QLQ-C30	**Symptom scale**			
	Dyspnoea	16.7 ± 25.9; 0; (0 - 33.3)	9.3 ± 16.9; 0 (0 - 8.3)	0.269
	Pain	12.1 ± 18.5; 0 (0 - 16.7)	10.2 ± 18.2; 0 (0 - 16.7)	0.671
	Fatigue	27.6 ± 21.7; 22.2 (11.1 - 33.3)	21.0 ± 23.7; 11.1 (0 - 33.3)	0.093
	Insomnia	26.3 ± 30.4; 33.3 (0 - 33.3)	15.7 ± 26.6; 0 (0 - 33.3)	0.096
	Appetite loss	9.8 ± 20.7; 0 (0 - 0)	0.9 ± 5.5; 0 (0 - 0)	**0.019**
	Nausea/vomiting	4.9 ± 11.8; 0 (0 - 0)	1.9 ± 5.2; 0 (0 - 0)	0.374
	Constipation	18.6 ± 28.2; 0 (0 - 33.3)	10.2 ± 23.3; 0 (0 - 0)	0.102
	Diarrhea	22.5 ± 25.2; 33.3 (0 - 33.3)	13.0 ± 25.2; 0 (0 - 8.3)	**0.038**
	**Financial difficulty scale**	5.9 ± 15.1; 0 (0 - 0)	7.4 ± 21.0; 0 (0 - 0)	0.969
	**Function scale**			
	Physical	86.7 ± 15.7; 93.3 (73.3 - 100)	90.4 ± 15.4; 100 (86-7 - 100)	0.195
	Role	82.8 ± 22.7; 100 (66.7 - 100)	90.3 ± 18.6; 100 (83.3 - 100)	0.131
	Cognitive	82.4 ± 22.1; 83.3 (83.3 - 100)	89.4 ± 19.3; 100 (83.3 - 100)	0.075
	Emotional	76.8 ± 21.4; 83.3 (66.7 - 100)	79.4 ± 20.6; 83.3 (66.7 - 100)	0.587
	Social	76.0 ± 27.8; 75 (66.7 - 100)	76.9 ± 23.0; 75 (66.7 - 100)	0.852
	**Global health status**	70.5 ± 18.4; 75 (66.7 - 83.3)	74.8 ± 17.7; 83.3 (64.6 - 83.3)	0.309
QLQ-PR25	Urinary symptoms	28.9 ± 17.9; 75 (62.5 - 83.3)	26.4 ± 17.6; 77.1 (58.3 - 84.4)	0.642
	Incontinence aid	43.3 ± 26.0; 66.7 (33.3 - 66.7)	24.1 ± 29.0; 83.3 (66.7 - 100)	**0.035**
	Bowel symptoms	11.3 ± 17.0 (0 - 33.3)	8.3 ± 13.9; 0 (0 - 33.3)	0.509
	Treatment symptoms	24.4 ± 19.0; 16.7 (0 - 33.3)	21.3 ± 15.6; 11.1 (0 - 33.3)	0.772
	Sexually active	31.4 ± 28.2; 33.3 (0 - 50)	33.8 ± 28.5; 33.3 (16.7 - 50)	0.709
	Sexual functioning	49.3 ± 20.8; 54.2 (45.8 - 66.7)	48.7 ± 16.6; 41.7 (33.3 - 66.7)	0.979

Values are displayed are [mean ± SD; median (IQR)] (IQR, interquartile range; SD, standard deviation; SLND, salvage lymph node dissection; SLNRT, salvage lymph node radiotherapy). Bold values represent p values <0.05.

Addressing continence outcomes, no statistical differences between the SLNRT and the SLND cohort were seen based on median daily pad usage [SLND: 1 (IQR 0.75 – 1.25); SLNRT: 1 (IQR 1 – 3); p=0.743] as well as median validated ICIQ-SF scores [SLND: 6 (IQR 0 – 8.3); SLNRT: 9 (IQR 4 – 10.8); p=0.072]. Furthermore, no statistically significant differences regarding median IIEF-5 scores were observed [SLND: 0.5 (IQR 0 – 5); SLNRT: 0 (IQR 0 – 2); p=0.160].

### Survival Outcomes

**Figure 1:**
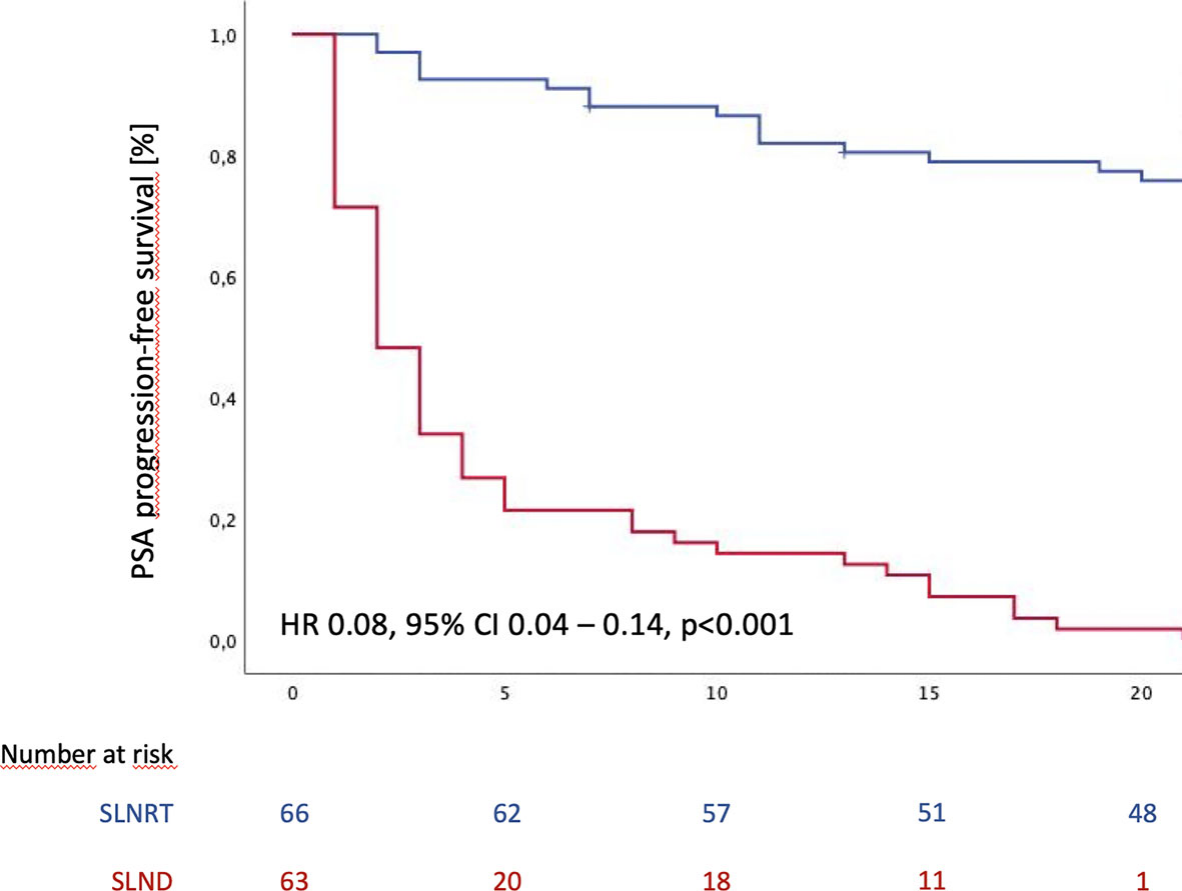
PSA progression-free survival following SLND (salvage lymph node dissection) and SLNRT (salvage lymph node radiotherapy) due to nodal recurrent prostate cancer CI, confidence interval; HR, hazard ratio).

At maximum follow-up, biochemical recurrence rate was 40.3% (27 patients) for the SLNRT cohort and 86.4% (57 patients; p<0.001) for the SLND cohort. As shown in **Figure 1**, estimated median PSA PFS was 65 months (95% CI 56.8 – 73.2) for the SLNRT cohort compared to 2 months (95% CI 1.3 – 2.7) for the SLND cohort (p<0.001). This difference in PSA PFS could be confirmed in further subgroup analyses after exclusion of patients with PSA persistence following salvage treatment (HR 0.08, 95% CI 0.04 – 0.14, p<0.001; **Supplementary Figure 1A**), patients who underwent pick-up SLND of one single lymph node only (HR 0.08, 95% CI 0.04 – 0.15, p<0.001; **Supplementary Figure 1B**), and patients who underwent SLND with removal of less than 5 lymph nodes (HR 0.13, 95% CI 0.06 – 0.28, p<0.001; **Supplementary Figure 1C**). Post-interventional PSA-persistence was observed in 18 SLNRT patients (26.9%) compared to 37 SLND patients (52.1%; p=0.003).

For univariate as well as multivariable Cox regression analysis, we accounted for differences in pre-interventional patient characteristics (**Table 1**) and stratified for treatment modality, ADT anytime during follow-up, pathological features at RP including positive surgical margins, pT stage and pN stage, as well as time interval between RP and salvage treatment, and PSA level at time of salvage treatment. Hereby, SLNRT could be confirmed as an independent predictor of increased PSA PFS (HR 0.08, 95% CI 0.040 – 0.142, p<0.001). Detailed results of univariate and multivariable analysis are summarized in **Table 3**.

**Table 3:** Univariate analysis and multivariate Cox regression model for PSA progression-free survival (ADT, androgen deprivation therapy; CI, confidence interval; RP, radical prostatectomy; SLND, salvage lymph node dissection; SLNRT, salvage lymph node radiotherapy).

Variable	Univariate			Multivariate		
	Hazard ratio	95% CI	p	Hazard ratio	95% CI	p
SLNRT at salvage treatment	0.08	0.042 - 0.141	<0.001	0.08	0.040 - 0.142	<0.001
ADT anytime during follow-up	0.51	0.299 - 0.866	0.013	0.98	0.572 - 1.703	0.962
Positive lymph nodes at RP	1.25	0.786 - 1.972	0.351	–	–	–
Positive surgical margin at RP	1.46	0.938 - 2.282	0.093	–	–	–
>=pT3 at RP	1.39	0.841 - 2.303	0.199	–	–	–
Time interval between RP and salvage treatment	1.00	0.995 - 1.005	0.933	–	–	–
PSA level at salvage treatment	1.03	1.000 - 1.054	0.048	1.02	0.990 - 1.056	0.181

**Figure 2:**
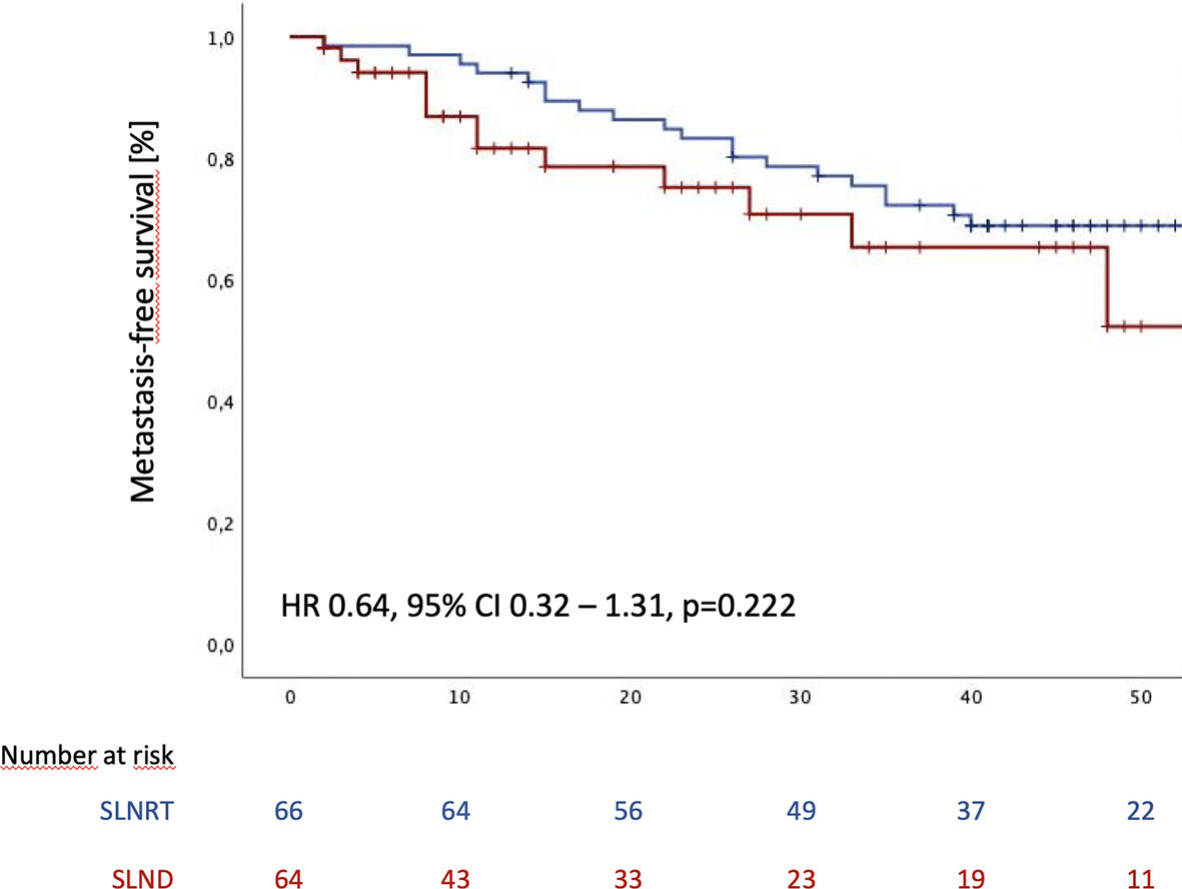
Metastasis-free survival following SLND (salvage lymph node dissection) and SLNRT (salvage lymph node radiotherapy) due to nodal recurrent prostate cancer CI, confidence interval; HR, hazard ratio).

During the follow-up period, distant metastases were observed in 21 patients in the SLNRT cohort (31.3%), compared to 20 patients (36.4%; p=0.419) in the SLND subcohort. Estimated median MFS was 70 months for the entire cohort (95% CI 34.1 - 105.9). In addition, estimated mean metastasis-free survival (MFS) was 57.6 months (95% CI 51.4 – 63.8) for SLNRT patients and 39.5 months (33.4 – 45.6) for SLND patients (**Figure 2**; HR 0.64, 95% CI 0.32 – 1.31, p=0.216).

## Discussion

The optimal management of patients with PSMA-PET/CT-based nodal-only recurrence of prostate cancer following RP is still controversial and mainly based on retrospective data. Recently, Bravi et al. reported long-term outcomes of a cohort of 189 patients with nodal-only recurrence after RP who underwent SLND between 2004 and 2011 (5). The authors found a 10yrs-BRFS of 11%, with 145 of 189 patients receiving ADT during the follow-up period and a median time to ADT of 41 months. In addition, freedom of cancer-specific death was 66%. Interestingly, patients who received ADT within 6 months from SLND had a significantly lower risk of cancer-related death in multivariate analysis (p=0.010). Based on these findings, the authors argued against the value of metastasis-directed therapy as a monotherapy for lymph node-only recurrent PC (5). In line with these contemporary findings, we found a median PSA PFS of 4.8 months with a PSA persistence rate of 52.8% in our SLND cohort. However, it has to be emphasized that a fair proportion of patients underwent pick-up-SLND (18.3%) and less than half of the patients (38%) underwent bilateral SLND. This is particularly important with regard to recent findings from a single institution, that described superior results for SLND compared to standard ADT, with improved survival rates compared to our SLND cohort (15). In contrast to our cohort, the majority of these patients underwent concomitant ADT, most frequently bicalutamide 50mg for a total period of 3 months. In addition, all patients underwent bilateral SLND, which had been shown to be of potential additional oncologic value in a large multi-institutional study (16). Altogether, these findings underscore the hypothesis, that pick-up-SLND should not be regularly performed and that bilateral SLND in combination with short-term ADT provides at least increased mid-term recurrence-free survival outcomes compared to mono-therapeutic unilateral SLND as performed in our cohort.

With the benefit of SLND in question, the search for therapeutic alternatives is ongoing. Hereby, comparative analyses between SLND and SLNRT are still rare. In our preliminary results, we found significantly increased 2yr-BRFS rates for irradiated patients compared to patients that underwent SLND (92% *vs.* 30%, p=0.001) (7). However, these results were limited by differences in sample size and only short follow-up in the SLND cohort. Boeri et al. compared a total number of 191 SLND patients to 63 patients that underwent SLNRT and found no statistically significant differences regarding recurrence-free survival as well as cancer-specific survival between both subgroups (15). Again, one must keep in mind that these patients underwent SLND in a multimodal therapeutic approach and that ADT was administered in the majority of SLND patients. The vast majority in the current SLND cohort did not undergo simultaneous ADT so no general conclusions regarding SLND in a multimodal approach can be drawn from this cohort. However, there are indications that SLND might be inferior as a monotherapy compared to a multimodal approach including SLNRT. Furthermore, the patient cohort described by Boeri et al. has been diagnosed with nodal recurrent PC based on conventional imaging or Choline-PET. In the current ^68^Ga-PSMA-based nodal recurrent cohort, we observed statistically significant increased biochemical recurrence rates for SLND compared to SLNRT patients (86.4 *vs.* 40.3%; p<0.001) and SLNRT as an independent predictor of increased BRFS was confirmed in multivariable analysis. In contrast, no statistically significant differences regarding MFS between both subgroups were observed. However, the differences between median follow-up in both subgroups have to be acknowledged with significantly longer follow-up in SLNRT patients (47 *vs.* 33 months). In addition, ADT concomitantly with SLNRT was applied in the vast majority of SLNRT patients for a median duration of 9 months. Nevertheless, at last follow-up only 13 patients in the SLNRT cohort (19.4%) compared to 43 patients (65.2%; p<0.001) in the SLND cohort were under active ADT treatment and SLNRT was confirmed as predictor for increased PSA PFS at last follow-up. Furthermore, as previously shown in our preliminary results, we observed significant differences in pre-interventional patient characteristics, tending towards a more aggressive disease in SLNRT patients (7).

With the oncological benefit of the aforementioned therapies still under debate, assessment of the impact on patient-reported outcomes and HRQOL is highly relevant. Since data on this important question is still lacking, we used various validated questionnaires in the current study to provide valid and generalizable results. Overall, we did not observe statistically significant differences in QLQ-C30 and QLQ-PR25 functioning scores. Regarding overall symptoms scores, increased rates in appetite loss and diarrhea could be observed for SLNRT patients based on the QLQ-C30 questionnaire. In contrast, the PR25 bowel symptoms score did not show significant differences between both subgroups. Regarding general HRQOL based on QLQ-C30 global health status, increased mean scores could be observed for SLND without reaching statistical significance. Similarly, no significant differences at the time of last follow-up could be observed for urinary incontinence and erectile dysfunction between both subgroups.

In addition to the implementation of patient-reported outcomes, the strengths of the study include the state-of-the-art PSMA-based staging, and the adequately large sample sizes. The current study is not devoid of limitations. In addition to the limitations that are inherent of the retrospective design of the current study, the limited follow-up in the SLND subgroup, the differences in ADT management, and the differences in baseline patients’ characteristics have to be addressed. Our HRQOL assessment is an important novelty in the current study. However, it has to be highlighted that no baseline HRQOL assessment has been performed and thus no longitudinal HRQOL analyses can be provided. Homogeneity of our cohort is increased by the fact that all included patients underwent RP as primary definitive treatment. Furthermore, no radio-guided surgical approach that showed promising results in small prospective cohorts has been performed in the current study (17).

It is important to emphasize that due to the heterogeneity of patient characteristics and periinterventional ADT management, no definitive conclusions regarding the superiority of one treatment modality over another can be drawn from the current analysis. Due to the fact that a majority of SLNRT underwent concomitant ADT, in contrast to SLND patients, we rather compare a multimodal SLNRT/ADT approach to a SLND monotherapy approach. However, we present real-life data from a large academic prostate cancer reference center that teach two important lessons with implications for future trial design. This is important to emphasize, since both techniques have still to be considered experimental that ultimately aim for delaying PSA progression and/or time to systemic therapy. Since patient characteristics between typical SLNRT and SLND intend to vary significantly based on our real-life experiences, adequate randomization strategies are mandatory. Furthermore, implementing our results into the existing literature indicates that SLND might not be sufficient as a monotherapy to provide adequate prolongation in PSA PFS. Even though we did not observe a statistically significant difference in MFS, this might be considered for future trial design (15).

## Conclusions

In summary, this is the largest study of contemporary patients that underwent salvage therapies due to PSMA-PET/CT-based nodal recurrent PC with assessment of oncological as well as patient-reported outcomes. While superior BRFS can be shown for SLNRT combined with short-term ADT compared to SLND as salvage therapy strategy, no differences in MFS and patient-reported outcomes including HRQOL could be observed.

## Data Availability Statement

The raw data supporting the conclusions of this article will be made available by the authors, without undue reservation.

## Ethics Statement

The studies involving human participants were reviewed and approved by Ethics committee of University Hospital Munich, Faculty of Medicine, LMU Munich. The patients/participants provided their written informed consent to participate in this study.

## Author Contributions

AK and N-SS-H contributed to conception and design of the study. AK, N-SS-H, and JM organized the database. AK, N-SS-H, and AB performed the statistical analysis. AK and N-SS-H wrote the first draft of the manuscript. All authors contributed to the article and approved the submitted version.

## Conflict of Interest

The authors declare that the research was conducted in the absence of any commercial or financial relationships that could be construed as a potential conflict of interest.

## Supplementary Material

Supplementary Figure 1 Subanalysis of PSA progression-free survival following SLND (salvage lymph node dissection) and SLNRT (salvage lymph node radiotherapy) due to nodal recurrent prostate cancer, excluding PSA-non-responders in both subgroups **(A)**, patients who underwent pick-up SLND of one single lymph node only **(B)**, and excluding patients who underwent SLND with removal of less than 5 lymph nodes **(C)**.
