# Supplementary figures and images for: Patient-Reported and Oncological Outcomes of Salvage Therapies for PSMA-Positive Nodal Recurrent Prostate Cancer: Real-Life Experiences and Implications for Future Trial Design
Source: Front Oncol. 2021 Jun 21;11:708595. doi: 10.3389/fonc.2021.708595 (PMC8255992; doi:10.3389/fonc.2021.708595)

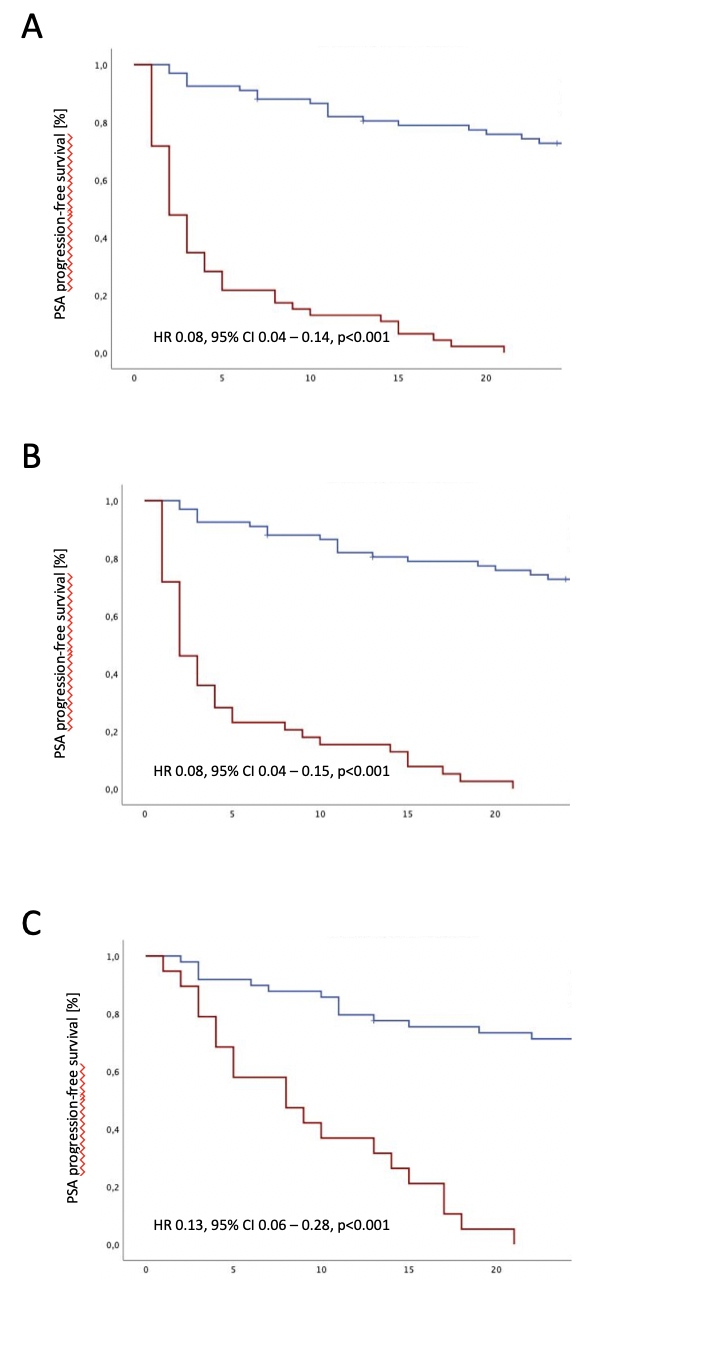

Supplement: Supplementary Figure 1 — Subanalysis of PSA progression-free survival following SLND (salvage lymph node dissection) and SLNRT (salvage lymph node radiotherapy) due to nodal recurrent prostate cancer, excluding PSA-non-responders in both subgroups (A), patients who underwent pick-up SLND of one single lymph node only (B), and excluding patients who underwent SLND with removal of less than 5 lymph nodes (C). [file Image_1.jpeg]
