# Supplementary material for: Patient-Reported and Oncological Outcomes of Salvage Therapies for PSMA-Positive Nodal Recurrent Prostate Cancer: Real-Life Experiences and Implications for Future Trial Design
Source: Front Oncol. 2021 Jun 21;11:708595. doi: 10.3389/fonc.2021.708595 (PMC8255992; doi:10.3389/fonc.2021.708595)
Supplement: Supplementary file 2 [file Table_1.docx]

| **Clavien** | **n** | **Detailed information** |
| --- | --- | --- |
| 1 | 0 |  |
| 2 | 7 | 3x lymphorrhea, 2x paralytic ileus, 1x hematoma, 1x asymptomatic hydronephrosis |
| 3a | 6 | 5x symtomatic lymphocele with percutaneous drainage, 1x thrombosis V. femoralis |
| 3b | 1 | Mechanic ileus, surgical revision |
| 4 | 2 | 2x pulmonary artery embolism |
| 5 | 0 |  |

**Supp. table 1.** Perioperative complications following salvage lymph node dissection based on the Clavien-Dindo scale (15).
