# Supplementary material for: Patient-Reported and Oncological Outcomes of Salvage Therapies for PSMA-Positive Nodal Recurrent Prostate Cancer: Real-Life Experiences and Implications for Future Trial Design
Source: Front Oncol. 2021 Jun 21;11:708595. doi: 10.3389/fonc.2021.708595 (PMC8255992; doi:10.3389/fonc.2021.708595)
Supplement: Supplementary file 3 [file Table_2.docx]

| **Toxicity** | **Acute toxicity** | | | **Late toxicity** | | |
| --- | --- | --- | --- | --- | --- | --- |
|  | **n (%)** | | | **n (%)** | | |
|  | **Grade 2** | **Grade 3** | **Grade 4** | **Grade 2** | **Grade 3** | **Grade 4** |
| **GU** | **19 (28%)** | **1 (2%)** | **-** | **17 (25%)** | **1 (2%)** | **-** |
| **GI** | **19 (28%)** | **-** | **-** | **1 (2%)** | **-** | **-** |
| **Other (erectile dysfunction)** |  |  |  | **6 (9%)** | **24 (36%)** |  |
| **GU: genitourinary; GI: gastrointestinal** | | | | | | |

**Supp. table 2.** Acute and late toxicity following salvage lymph node radiotherapy.
